# Fine particulate matter (PM_2.5_) in China at a city level

**DOI:** 10.1038/srep14884

**Published:** 2015-10-15

**Authors:** Yan-Lin Zhang, Fang Cao

**Affiliations:** 1 Yale-NUIST Center on Atmospheric Environment, Nanjing University of Information Science and Technology, Nanjing 10044, China

## Abstract

This study presents one of the first long term datasets including a statistical summary of PM_2.5_ concentrations obtained from one-year monitoring in 190 cities in China. We found only 25 out of 190 cities could meet the National Ambient Air Quality Standards of China, and the population-weighted mean of PM_2.5_ in Chinese cities are 61 μg/m^3^, ~3 times as high as global population-weighted mean, highlighting a high health risk. PM_2.5_ concentrations are generally higher in north than in south regions due to relative large PM emissions and unfavorable meteorological conditions for pollution dispersion. A remarkable seasonal variability of PM_2.5_ is observed with the highest during the winter and the lowest during the summer. Due to the enhanced contributions from dust particles and open biomass burning, high PM_2.5_ abundances are also found in the spring (in Northwest and West Central China) and autumn (in East China), respectively. In addition, we found the lowest and highest PM_2.5_ often occurs in the afternoon and evening hours, respectively, associated with daily variation of the boundary layer depth and anthropogenic emissions. The diurnal distribution of the PM_2.5_-to-CO ratio consistently displays a pronounced peak during the afternoon periods, reflecting a significant contribution of secondary PM formation.

Due to a rapid economic development, industrial expansion and urbanization during the last few decades in China, increasingly occurrence of haze or smog episodes characterized by the high fine particulate matter (i.e. with aerodynamic diameters not larger than 2.5 μm, or PM_2.5_) levels and reduced visibility has been reported in national-scale China especially in the most developed and high-populated city clusters such as the Yangtze River Delta (YRD), the Pearl River Delta (PRD) and the Beijing-Tianjin-Hebei (BTH) regions^1^,^2^,^3^,^4^,^5^,^6^,^7^. Urban PM_2.5_ originates mainly from sources such as traffic-related emissions, road/soil dust, biomass burning, and agriculture activities as well as regional transported aerosols, but it still remains much challenging to quantify contributions from each source and understand PM formation mechanism^2^,^7^,^8^,^9^,^10^,^11^. According to a report from Asian-development Back, only <1% of 500 largest cities of China could meet the air quality guideline (10 μg/m^3^ for annual mean and 25 μg/m^3^ for 24-hour mean) suggested by the World Health Organization and several cities are ranked among the most polluted cities in the world^12^.

High occurrence of extreme haze episodes in recent years not only leads to a global concern due to its adverse health effects^13^,^14^, but also triggers the Chinese government to tackle the serious air quality problem especially PM_2.5_ pollution. From February 29th, 2012, the Chinese Ministry of Environmental Protection (MEP) published the third revision of the “the national ambient air quality standards” (NAAQS) (GB3095-2012), in which PM_2.5_ is included into the NAAQS for the first time. PM_2.5_ monitoring has not yet been introduced in the national network in China before the new NAAQS, although PM_2.5_ levels have been reported in research studies in some developed cities such as Beijing, Shanghai, Chongqing and Guangzhou^7^,^8^,^15^,^16^,^17^,^18^. Due to the lack of large-scale PM_2.5_ monitoring, the spatial distribution of urban PM_2.5_ in China is often retrieved by the satellite remote sensing^19^. Large scale real-time PM_2.5_ mortaring is very necessary to study spatial and temporal (i.e. seasonal and diurnal) variation of urban PM_2.5_ in China. Wang *et al.* (2014) reported a study on the spatial and temporal variations of the 6 criteria pollutants in 31 provincial capital cities in China during 2013–2014 20. The study reveals PM_2.5_, PM_10_, CO and SO_2_ concentrations are higher in the North region than those in the West and the South-East regions, although high pollution events are also frequently observed during the autumn for the South-East region and during the spring for the West region. However, little has been reported regarding diurnal patterns in aerosol concentrations in different seasons due to the lack of real-time monitoring. In addition, a higher spatial-temporal resolution is also needed to obtain a better understanding of air quality problem at a city-level.

This paper presents one of the first long term data sets including a statistical summary of PM_2.5_ concentrations measured during one-year continuous monitoring in 190 priority pollution monitoring cities in China. A national air quality monitoring network with nearly 950 monitoring stations in 190 Chinese cities is put into operation by the end of 2013, which release real-time monitoring data on air quality such as PM_2.5_, PM_10_, SO_2_, NO_2_, CO, O_3_ to the public. In this study, we will present an overview of spatial and seasonal distribution of PM_2.5_ in 190 cities of China. In addition, diurnal variation of PM_2.5_ in the most populated and developed cities of the PRD, YRD and BTH regions will be discussed. Finally, the population-weighted mean of PM_2.5_ and cumulative population distribution in Chinese cities will be estimated. By studying this extensive dataset at a national-scale level, we could improve our understanding of urban PM_2.5_ pollution at a fine spatiotemporal resolution in China. The obtained knowledge in this study is therefore very useful for the implementation of national/regional air pollution reduction policy in order to meet Chinese new NAAQS.

## Results Overview and spatial distributions of PM_2.5_ pollution in China

**Figure 1:**
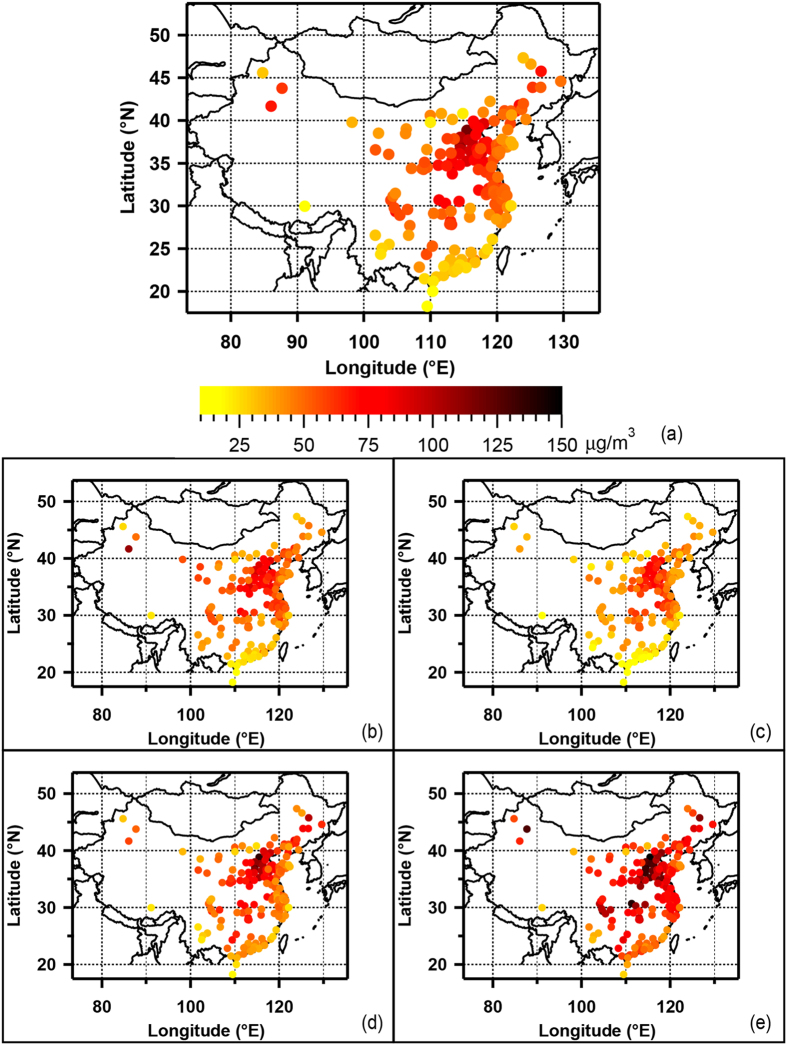
The averaged PM_2.5_ concentrations (μg/m^3^) of the 190 cities of China, during the year of 2014/2015 (a) and during the spring (b), summer (c), autumn (d), and winter (e). The maps were drawn by the software of Igor Pro, http://www.wavemetrics.com/.

Figure 1a shows spatial distributions of annual mean of PM_2.5_ concentrations of 190 cities in China. The new NAAQS of China sets PM_2.5_ concentration limits for both the 24-hour average (35 μg/m^3^ and 75 μg/m^3^ for Grade I and II, respectively) and the annual mean value (15 μg/m^3^ and 35 μg/m^3^ for Category I and II zones, respectively)^21^. The annual 24-h PM_2.5_ ranges from 18 μg/m^3^ (in Sanya) to 116 μg/m^3^ (in Baoding) with average of 57 ± 18 μg/m3, severely exceeding the NAAQS of China and other standards recommended by international organizations and other countries (10–35 μg/m^3^)^21^,^22^,^23^. According to the new NAAQS (Grade II), as many as 165 cities cannot meet the standards, accounting for 87% of the total number of cities. PM_2.5_ concentrations are generally higher in the cities located in the North regions than those observed in the South regions. PM_2.5_ concentrations also tend to be lower in the coastal regions than in the inland regions. As can be seen in Fig. 1a, the highest annual average concentration is observed in the Beijing-Tianjin-Hebei (BTH) region including two megacities of Beijing and Tianjin and Hebei Province, which has the highest density of coal consumption and heavy industries (e.g. iron, steel and cement) in China. It is important to note that half of the 10 worst haze affected cities are in Beijing-Tianjin-Hebei, namely Baoding, Xingtai, Shijiazhuang, Handan and Hengshui (see Table S1). Several studies have revealed that the enhanced PM pollution in BTH is not only due to the primary emissions from local sources such as industrial and domestic and agricultural sources but also due to the regional transported contribution (e.g. from nearby Shandong and Henan Provinces) and secondary production^8^,^9^,^17^,^18^,^24^,^25^. Furthermore, the climate of BTH is characterized by stagnant weather with weak wind and relatively low boundary layer height, leading to the favorable atmosphere conditions for accumulation, formation and processing of aerosols^26^. The lowest PM_2.5_ concentration is observed in Hainan Province, the southernmost island of China, which is due to the less anthropogenic emissions and favorable meteorological condition for atmospheric dispersion and dilution. Due to the less coal-based industries and good dispersion weather conditions, the PM_2.5_ concentrations in PRD (i.e. cities in Guangdong province, see Table S1) are generally lower than those in the other two largest city clusters in China such as BTH and YRD (cities in Jiangsu and Zhejiang provinces as well as Shanghai, see Table S1), although 7 out of 21 cities in PRD still could not meet the new NAAQS of China.

## Seasonal variation of PM_2.5_ pollution in China

**Figure 2:**
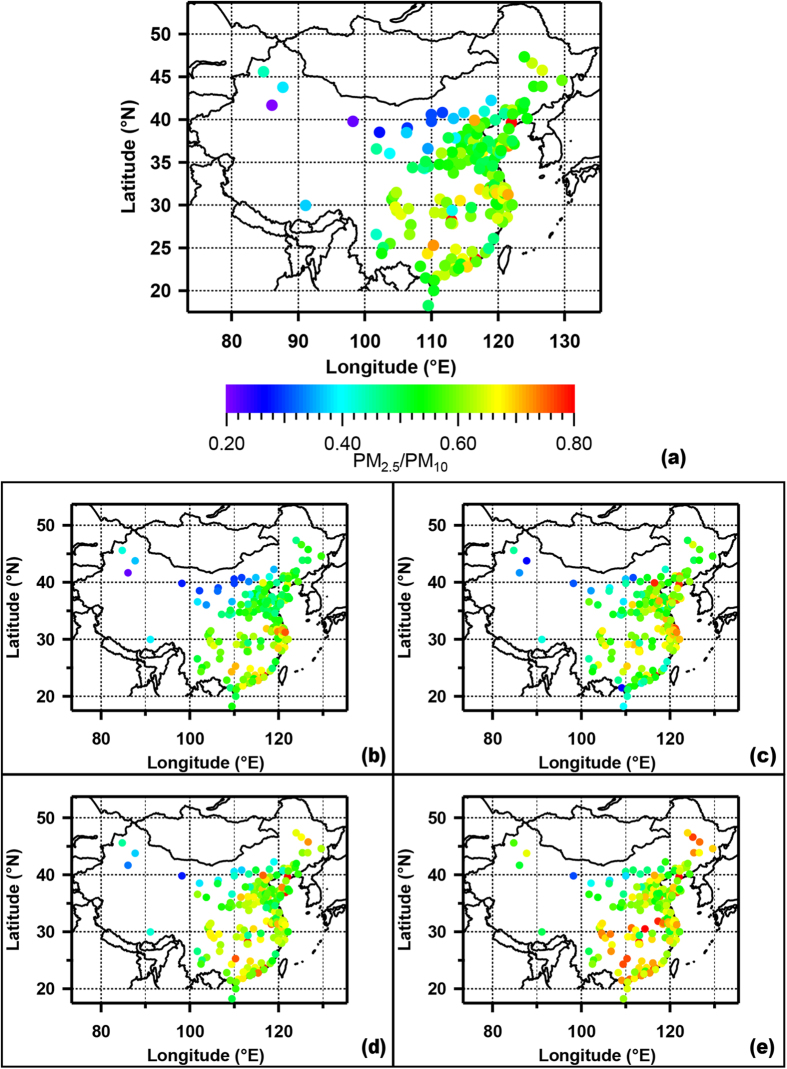
The averaged PM_2.5_/PM_10_ ratio of the 190 cities of China, during the year of 2014/2015 (a) and during the spring (b), summer (c), autumn (d), and winter (e). The maps were drawn by the software of Igor Pro, http://www.wavemetrics.com/.

Generally, PM_2.5_ concentrations show a remarkable seasonal variability with the highest during the winter and the lowest during the summer (Fig. 1). The wintertime maximum is associated with enhanced anthropogenic emissions from fossil fuel combustion and biomass burning and unfavorable meteorological conditions (i.e. more frequent occurrences of stagnant weather and temperature inversion during the cold periods) for pollution dispersion. In addition to accumulation of primary emissions, new particle formation and secondary production of both the inorganic aerosols and organic matters could further enhance fine PM abundance^8^,^9^. Actually, the PM_2.5_ to PM_10_ ratio is slightly higher during the winter than during the other seasons (Fig. 2 and S1), suggesting importance of combustion sources and secondary formation of fine particles. However, the seasonality displays a spatial variability. For Northwest and West Central China, the most polluted season appears in spring but not in winter, associated with increased contribution from dust particles in this desert‐like region. PM_2.5_/PM_10_ ratio (i.e.0.21) during the spring in Korla (41.7N, 86.1E), one of most polluted city in West China, are much lower than all other studied cities in China (i.e. with a mean of 0.56 ± 0.10). The decrease of PM_2.5_ to PM_10_ ratios are also observed in many cities in West Central and Northwest China (Fig. 2). The higher abundance of the coarse particles (PM_2.5–10_) in PM_10_ indicates a significant contribution from local dust emission and regional dust transport, as the other coarse-mode particle such as biogenic-derived large particles (e.g. plant debris) could only have a minor if not a negligible contribution with its cold desert climate and low vegetation coverage. Actually, the dust storm is most frequently occurred in the west regions in Northern China in spring from recent observations where desert, semi-desert or grassland is distributed^27^. This is also evident by seasonal variation of PM_2.5_/PM_10_ ratios (Fig. 2 and S2), suggesting significant importance of dust particles to PM_2.5_ in west regions of China. The substantial fraction of PM_10_ mass in the PM_2.5–10_ size range also suggests that the current PM_2.5_ control strategies (i.e. reduce fossil/non-fossil combustion derived VOCs and PM emissions) will only partly reduce the PM_10_ pollution in the west part of China. PM_2.5_ is much decreased during summer associated with the reduced anthropogenic emissions such as fossil fuel and biomass burning for domestic heating. Further, large wet depositions of aerosols, clean air mass from ocean during the Asian summer monsoon and enhanced convection within a higher atmospheric mixing layer could lead to a strong dilution effect of aerosols in Eastern China^28^. As a result, the PM_2.5_ minima are observed in summer. Interestingly, relatively high PM_2.5_ levels are also found during the autumn over Eastern China, which is likely due to enhanced open biomass burning during the agricultural harvest season. The fire counts map derived from MODIS on the NASA satellite (Figure S2) shows that during the October, intensive open biomass burning (i.e. agricultural waste burning) events occur over Northeast and South China. It should be also noted that PM_2.5_ concentrations during the autumn in the cities of the PRD and Northeast regions are higher than those found during the spring. In few cases (9 out of 190 cities), PM_2.5_ is even higher during the autumn than during the winter. The importance of biomass burning contributions to PM_2.5_ during autumn over PRD and Northeast China has also been underscored by previous studies^29^,^30^. For example, Wang *et al.* (2007) reported up to 19% of total PM_2.5_ is from biomass burning emissions in Guangzhou, the largest city in the PRD region^31^.

## Diurnal variation of PM_2.5_

**Figure 3:**
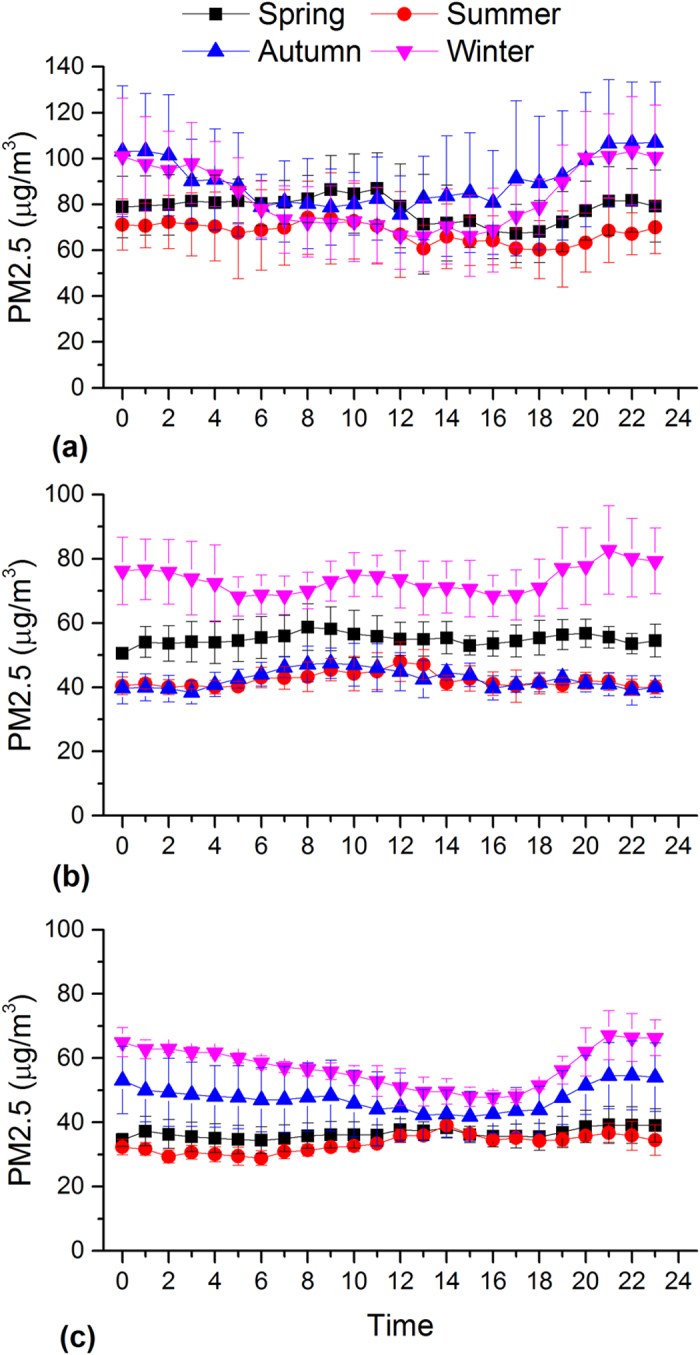
Diurnal variations of hourly PM_2.5_ concentrations in Beijing (a), Shanghai (b) and Guangzhou (c).

In this study, hourly data were used to examine diurnal variability in PM_2.5_ as well as the other major air pollutants. This provides importance information in identifying the potential emission sources and the time when the pollution level exceeds the standards. Figure 3 illustrates the diurnal variation of hourly PM_2.5_ concentrations in each season in Beijing, Shanghai and Guangzhou, the largest megacities in BTH, YRD and PRD in different climatic zones of China, respectively.

**Figure 4:**
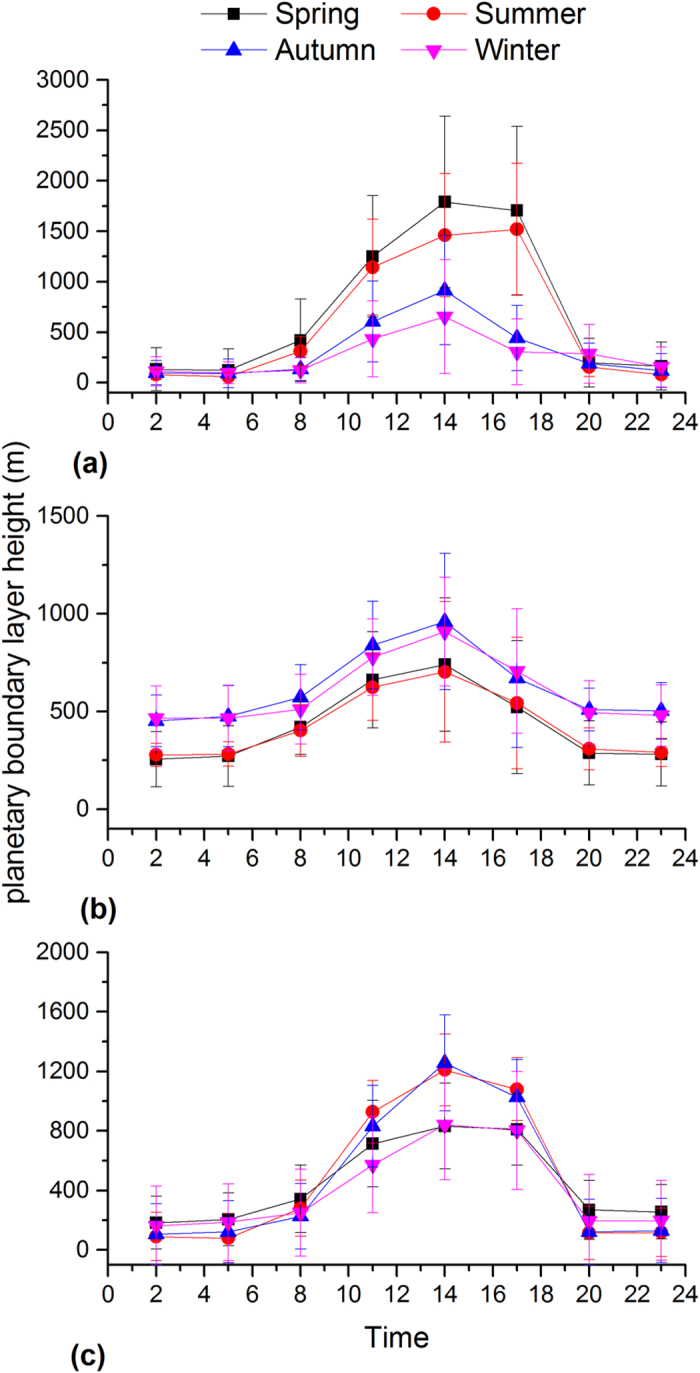
Daily evolution of average planetary boundary layer height in Beijing (a), Shanghai (b) and Guangzhou (c).

**Figure 5:**
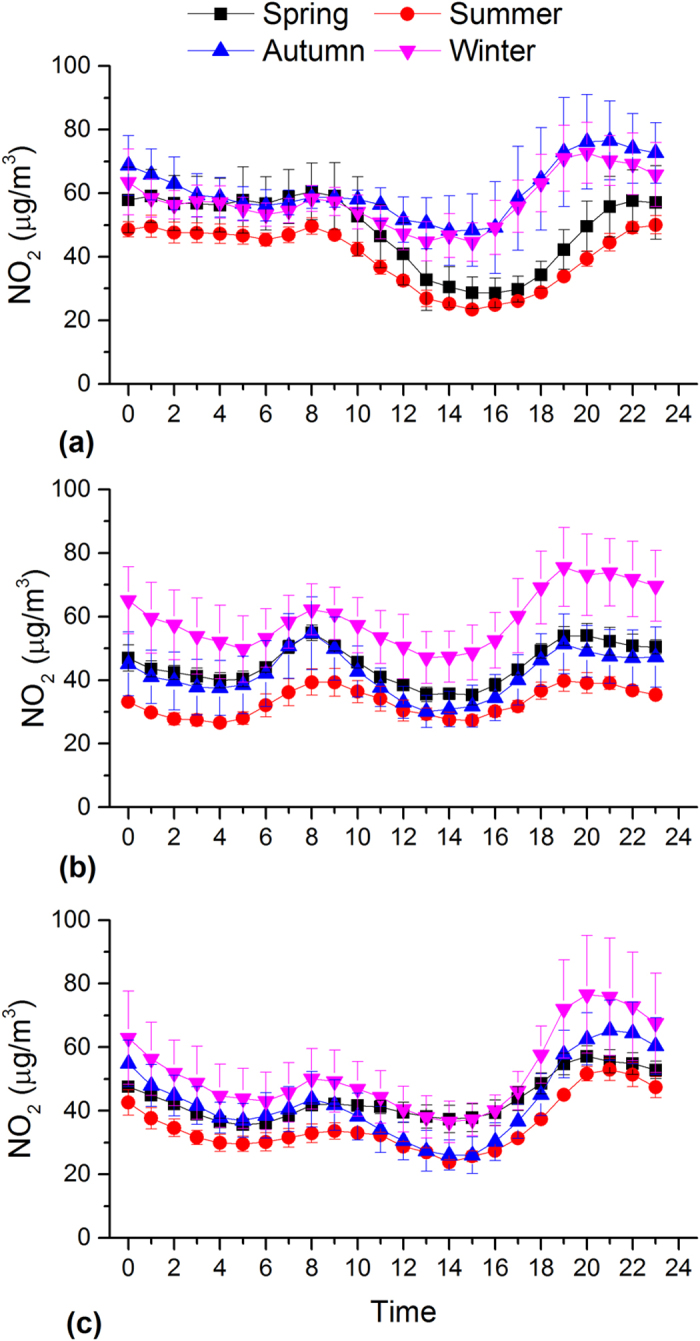
Diurnal variations of hourly NO_2_ concentrations in Beijing (a), Shanghai (b) and Guangzhou (c).

**Figure 6:**
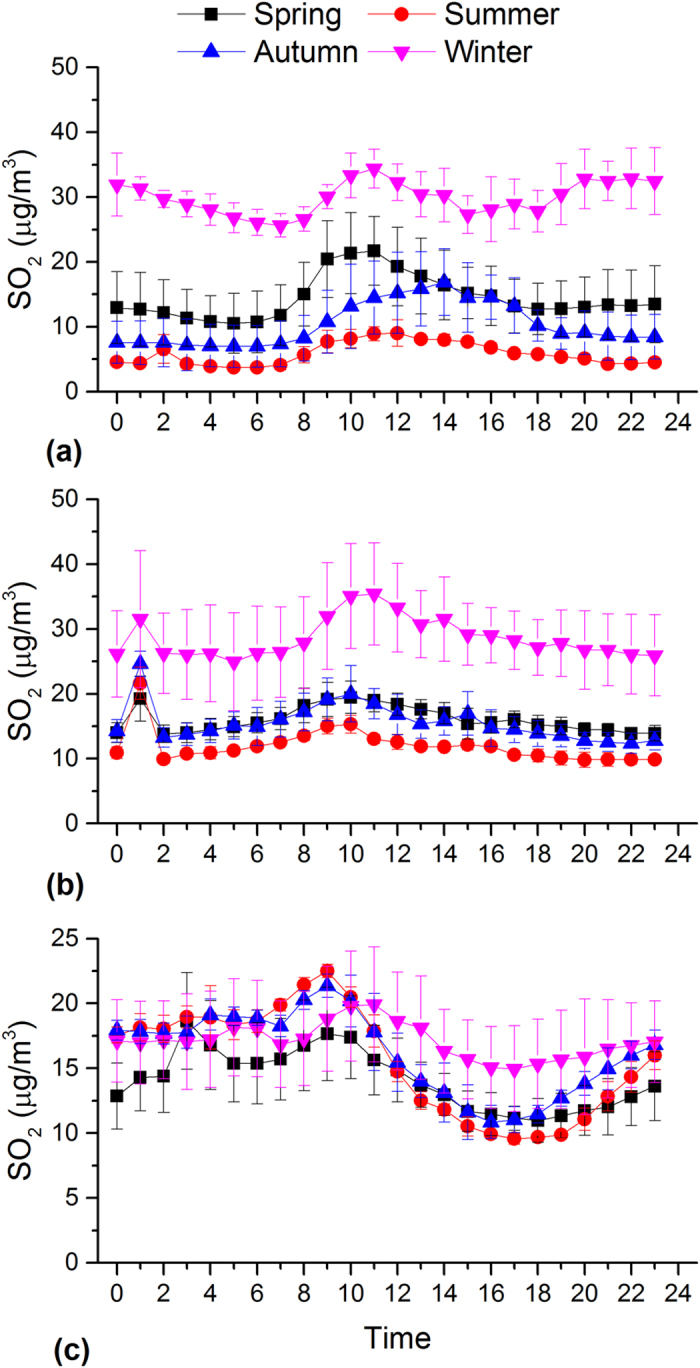
Diurnal variations of hourly SO_2_ concentrations in Beijing (a), Shanghai (b) and Guangzhou (c).

**Figure 7:**
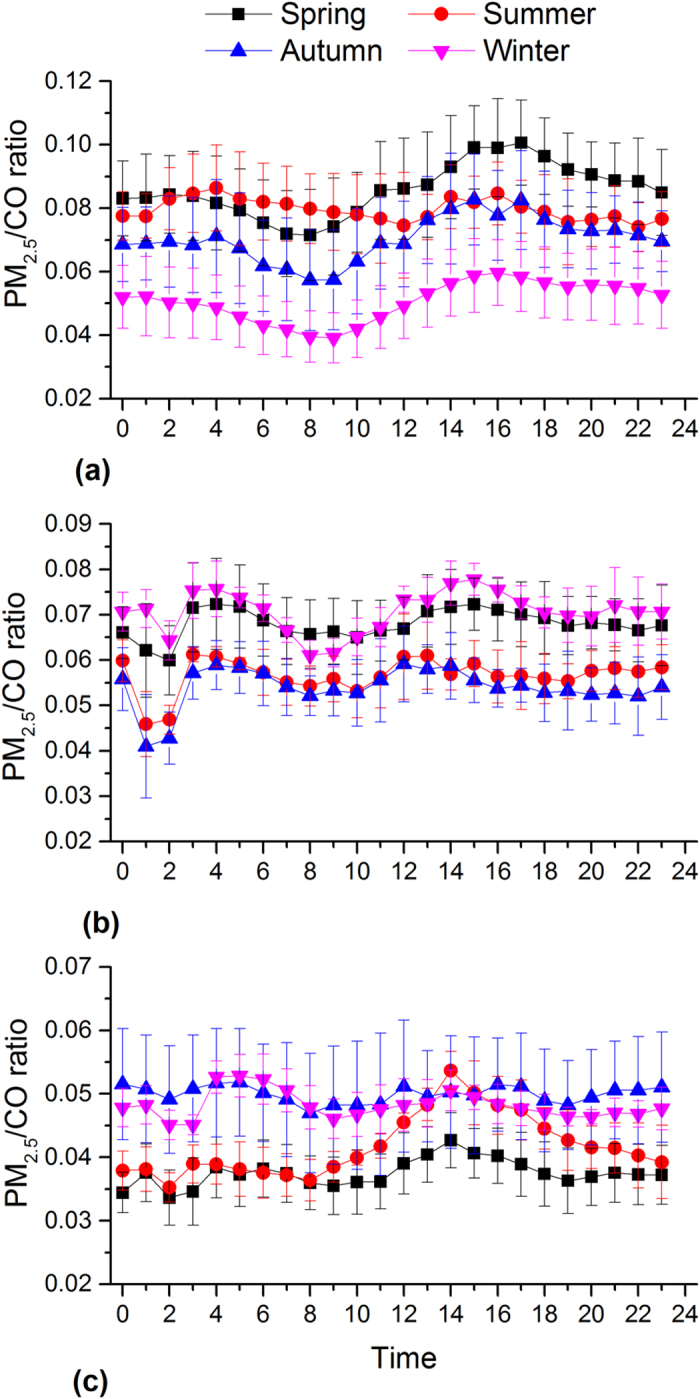
Diurnal variations of hourly PM_2.5_ to CO ratios (PM_2.5_/CO) concentrations in Beijing (a), Shanghai (b) and Guangzhou (c).

In Beijing, PM_2.5_ shows significantly higher concentrations and pronounced diurnal variations during the autumn and winter than during the spring and summer (Fig. 3a). The PM_2.5_ concentration during the autumn and winter is higher during the nighttime than the daytime, which can be explained by the enhanced emission for heating and relatively low the boundary layer (Fig. 4). The peak PM_2.5_ concentration at night is ~2 times higher than that of valley in the afternoon. The lowest concentrations are observed in afternoon hours when the boundary layer becomes larger and wind speed increases (data not shown). After 16:00, PM_2.5_ concentrations start to increase because of the quickly decreasing boundary layer heights (Fig. 4) and increasing vehicle emissions as evident by increased NO_2_ emissions (see Fig. 5). Moreover, PM pollution emitted from diesel truck traffic which is allowed only during nighttime additionally increase PM burden because emission factors of heavy-duty vehicles are 6 times than those from light-duty vehicles^32^. It should be noted that such a traffic restriction has been applied in many Chinese megacities which may affect diurnal pattern of PM_2.5_ and its chemical compositions. PM_2.5_ levels in afternoon periods during winter are on average even lower than those observed during the other seasons. Such a trend is not observed for other major air pollutants such as NO_2_ and SO_2_ (Figs 5 and 6). This indicates that the PM_2.5_ levels in Beijing are not only driven by primary emissions but are also affected by other factors such as meteorological conditions and secondary PM production. To further discuss secondary production of PM_2.5_, CO is used to normalize PM_2.5_ concentrations to exclude the influence of primary combustion emissions and meteorological factors^33^. Figure 7 shows diurnal variation of hourly ratios of PM_2.5_ to CO (i.e. an excellent tracer for primary combustion source). A pronounced peak of the PM_2.5_/CO ratio occurs during the afternoon (i.e. abound 16:00) for all the seasons although the ratio is apparently lower during the winter than during the other seasons. This indicates secondary formations process also plays an important role in PM concentrations, especially in the afternoon when the photochemical activities are relatively strong. However, a more detailed explanation is subject to further studies including more comprehensive observations of chemical compositions of PM_2.5_ and its precursors as well as meteorology. In spring and summer, the moderate PM_2.5_ peak appears in the morning (i.e. 8:00 am to 11:00 am), in accordance with high NO_2_ emissions from transportation during rush hours.

As shown in Fig. 3b, a unique diurnal pattern is observed in Shanghai. PM_2.5_ concentration during the winter is higher than those found in the other seasonal throughout the day, which could be explained by the enhanced anthropogenic emissions. In addition, the heavily polluted air mass is often transported from Northern China to the YRD region under East Asian winter monsoon climate (Figure S3), affecting the regional air quality in YRD^34^. The enhancement of SO_2_ concentration in the winter is comparably higher than that of NO_2_ (Figs 5 and 6), suggesting the importance of coal combustion emissions (i.e. power plant). There are often two moderate peaks of PM_2.5_ concentrations: one in the morning between 7 and 10 am, and another in the evening between 7 and 10 pm. A similar morning time peak is also found for both SO_2_ (i.e. a tracer for coal combustion) and NO_2_ (i.e. a tracer for vehicle emissions), suggesting a significant contribution of fossil emissions (e.g. from power plant and vehicle exhaust) to daytime PM_2.5_. Concentrations decrease from mid-morning to later afternoon/early evening due to a combination of the increasing boundary layer depth and reduced anthropogenic emissions. The afternoon increased PM_2.5_ is consistence with observation of NO_2_, suggesting traffic-related emissions may play a more important role in the PM_2.5_ variation from the afternoon to the evening hours. The diurnal distributions of PM_2.5_/CO show lowest levels during the evening and morning hours suggesting a predominance of combustions in the rush (traffic) hours.

Similar to Beijing, a strong daily variation of PM_2.5_ in Guangzhou is found during the autumn and winter, which is characterized by the minima in the late afternoon and maxima in the night. During the autumn and winter, an increase in PM_2.5_ is not observed during the morning periods although both the SO_2_ and NO_2_ are increased due to the increased anthropogenic emissions. Alternatively, this decreasing may be the result of increasing boundary layer depth. In these two seasons, the PM_2.5_ levels start to increase from the later afternoon, which could be explained by the increased motor vehicle emissions as NO_2_ is also dramatically increased during the morning time. It is interesting to note that the daily variation pattern of SO_2_ (and NO_2_) is very similar in different seasons, although PM_2.5_ displays a remarkably different diurnal cycle during the autumn and winter compared to during the spring and summer. The results indicate that PM formation process is very complex and is not only determined by the emission strength but is also driven by the other influencing factors such as the meteorological conditions and atmospheric oxidant capacity. During the spring and summer, a moderate PM_2.5_ peak often occurs in the early afternoon, typically earlier than peak hours during the autumn and winter. This phenomenon (i.e. a shift of peak time in the afternoon) is likely due to the longer and stronger solar irradiation, increasing O_3_ concentrations and higher temperatures, leading to the enhanced photochemical formation of secondary aerosol particles, being important constituents of PM_2.5_. Secondary organic aerosols formation from biogenic volatile organic compounds during spring and summer may also increase PM_2.5_ levels because the Guangzhou is characterized by the sub-tropical climate with the annual mean temperature of around 25°C and high evergreen vegetation coverage^35^,^36^. The PM_2.5_ concentration keeps in high levels during the nighttime (i.e. ~19:00 to midnight) in all the seasons, associated with primary automobiles emissions and the subsequent accumulation and secondary PM production. The PM_2.5_/CO ratio show a moderate daily variability with the highest value in the afternoon time during the spring and summer, because of the longer and stronger solar irradiation and increasing O_3_ concentrations as discussed before. However, no significant diurnal pattern is observed for the other two seasons. It should be also noted that the PM_2.5_/CO ratio is generally lower than those in Beijing and Shanghai, indicating a more important contribution of combustion emissions (i.e., from gasoline vehicles and biofuel) to PM_2.5_ concentration in Guangzhou and/or higher contribution of coal combustions in Beijing and Shanghai.

## Discussion

This study analyzes PM concentration data collected from the newest air quality monitoring network of the Ministry of Environmental Protection of China in 190 major cities during April 2014 to April 2015. The annual averaged concentration of PM_2.5_ is 57 ± 18 μg/m^3^ (ranging from 16 to 119 μg/m3), severely exceeding the new NAAQS (35 μg/m^3^) of China and World Health Organization air quality guideline (10 μg/m^3^). PM_2.5_ concentrations are generally higher in the cities located in the north region than those observed in the south regions, and are lower in the coastal regions than in the inland regions. This suggests that different control strategies should be targeted in different regions of China in terms of specific feature of local/regional emissions and meteorology. Generally, PM_2.5_ concentrations show a remarkable seasonal variability with the highest during the winter and the lowest during the summer. The winter maximum PM_2.5_ level is due to the increasing anthropogenic activates such as fossil-fuel and biomass burning for heating in the cold season. Furthermore, more frequent occurrences of stagnant weather and temperature inversion during the cold period may facilitate the accumulation of air pollutants. Alternatively, more frequent occurrences of precipitation and mixing of transported clean air mass result in a strong deposition, dispersion and dilution of PM_2.5_ in summer. It should be also noted the most polluted season appears in spring but not in winter for some cities located in Northwest and West Central China, associated to a higher contribution from dust particles in this desert‐like region. Moderately high PM_2.5_ is also observed during the autumn due to the enhanced open biomass burning emissions in the harvest season. The diurnal pattern of PM_2.5_ concentration as well as other major air pollutants such as NO_2_, SO_2_ and CO is also characterized. In Beijing and Guangzhou, a pronounced daily variation of PM_2.5_ concentration is found during the autumn and winter, with the lowest levels in the afternoon hours, apparently due to the deeper boundary layer. The PM_2.5_ keeps in high abundance at the evening hours in the cold seasons because of increasing emissions for heating and stagnant atmospheric conditions. Finally, CO is used to normalize PM_2.5_ concentrations to exclude the influence of primary combustion emissions and meteorological factors. The diurnal distribution of the PM_2.5_ to CO ratio (i.e. PM_2.5_/CO) consistently displays a remarkable peak during the afternoon periods, reflecting a significant contribution of secondary particle formation due to the relatively stronger solar irradiation and higher temperature. Our results demonstrate that the spatial-temporal distribution of PM_2.5_ in China is determined by complex factors such as local/regional-transported emissions, meteorological conditions and atmospheric oxidant capacity. In order to obtain a more comprehensive picture of source and formation mechanism of fine particulate matter pollution over national wide China, the real-time chemical composition of PM_2.5_ and the major air pollutants (e.g. NOx, SO_2_, O_3_, CO and volatile organic compounds) as well as meteorological conditions should be simultaneously investigated in a fine spatial-temporal resolution in future.

**Figure 8:**
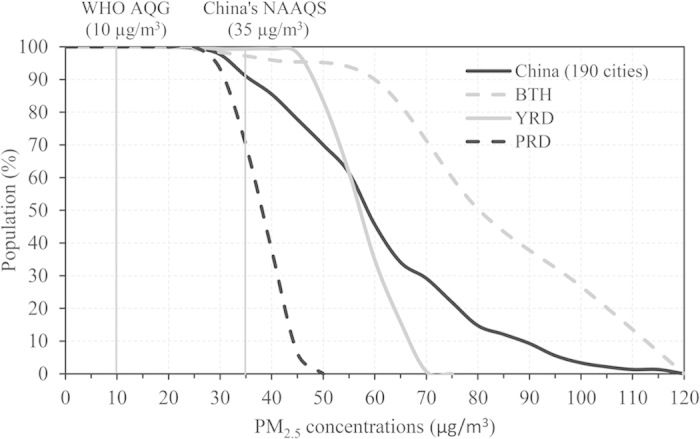
Cumulative distribution of annual mean PM_2.5_ estimated from ground measurements in 190 Chinese cities. The results from the Beijing-Tianjin-Hebei (BTH), Yangtze River Delta (YRD), and Pearl River Delta (PRD) regions are also displayed.

A major challenge for assessments of PM_2.5_-associated health impacts often lies in the lack of ground-based monitoring networks and representative exposure estimates^19^. This paper provides one of first datasets including PM_2.5_ levels in 190 Chinese cities from ~950 monitoring sites, which could be used for assessments of air pollution health impacts. Based on annual 24-hour PM_2.5_ and population data in each studied city, the population-weighted mean of PM_2.5_ in Chinese cities are 61 μg/m^3^, approximately 3 times as high as global population-weighted mean (20 μg/m^3^)^19^. Figure 8 shows the cumulative population distribution in Chinese cites as well as the most three developed regions such as the BTH, YRD and PRD regions. Overall, only 9% of the population live in cities with annual PM_2.5_ mean smaller than the new NAAQS in China (35 μg/m^3^). It should be noted that all the population live in cities where the WHO Air Quality Guideline (10 μg/m^3^) is exceeded. All studied regions show a nonlinear relationship of PM_2.5_ concentrations with population, which is consistent with the previous findings conduced in other places around the world^19^. The cities in the BTH region have the highest levels of PM_2.5_ concentrations, with 70% of the regional population exceeding 70 μg/m^3^ (twice as high as the NAAQS of China), whereas in the relatively clean region (i.e., PRD) there is still 70% of population residing in the cities exceeding the NAAQS of China (i.e., 35 μg/m^3^). Hoek *et al.* (2013) estimated that an increase of 10 μg/m^3^ in long-term PM_2.5_ exposure increases a 6.2% (95% confidence interval 4.1–8.4%) of all-cause mortality based on meta-analysis of cohort studies^37^. Taken together, this study highlights the significantly high health risk from fine particulate matter pollutions and difficulties in achieving air quality targets in China. Therefore, China needs much more effective policies and research that could mitigate complex PM_2.5_ pollutions, reduce urban population exposures, and achieve sustainable development. Future epidemiologic studies are urgently required to provide direct estimation of relationship of PM_2.5_ and mortality such as chronic cardiovascular and respiratory diseases in China and other polluted countries/regions.

## Methods

The national air quality monitoring network is continuously operated and maintained by Department of the Environment for each city. The network comprises of 496 stations in 74 cities since 2012, which is now extended to 946 monitoring stations in 190 cities (Table S1 and S2) from 2013. At each monitoring site, the real-time mass concentrations of PM_2.5_ and PM_10_ are measured using the micro oscillating balance method and/or the β absorption method from commercial instruments. Gas pollutants such as SO_2_, NO_2_, CO are measured using the ultraviolet fluorescence method, the chemiluminescence method and the gas filter correlation infrared absorption (or the non-dispersive infrared absorption method), respectively, by a set of commercial instruments. The instrumental operation, maintenance, data assurance and quality control are properly conducted according to the most revisions of China Environmental Protection Standards such as “HJ 193–2013^38^” and “HJ 655–2013^39^”. The real-time hourly PM_2.5_ concentrations data as well as other major air pollutant (i.e. SO_2_, NO_2_, PM_2.5_ PM_10_, CO, and O_3_) are continuously recorded by the MEP in China and are publicly accessible^40^. The data present in this study was obtained from the website during the period from 12-Apr-2014 to 11-Apr-2015. The hourly and 24-h (daily) concentrations of all the air pollutants were the averages of the hourly data from all monitoring sites in the city. Time-series (3-hour resolution) of planetary boundary layer depths were obtained from the U.S. National Oceanic and Atmospheric Administration (NOAA) READY archived meteorological GDAS data (1° × 1°) based on Coordinated Universal Time (UTC). All UTC values are converted to local time (Beijing time, UTC + 8). Statistical analyses are carried out using t-test (Excel 2010) when comparisons are made.

## Additional Information

**How to cite this article**: Zhang, Y.-L. and Cao, F. Fine particulate matter (PM_2.5_) in China at a city level. *Sci. Rep.* **5**, 14884; doi: 10.1038/srep14884 (2015).

## Supplementary Material

Supplementary Information
