# Supplementary material for: Fine particulate matter (PM2.5) in China at a city level
Source: Sci Rep. 2015 Oct 15;5:14884. doi: 10.1038/srep14884 (PMC4606739; doi:10.1038/srep14884)
Supplement: Supplementary Information [file srep14884-s1.pdf]

SUPPLEMENTARY INFORMATION

for

Fine particulate matter ($PM_{2.5}$) in China at a city level

Yan-Lin Zhang[1, *] and Fang Cao[1]

[1]Yale-NUIST Center on Atmospheric Environment, Nanjing University of Information Science and Technology, Nanjing10044, China

*Corresponding Author

Phone: 0086-25-58695681; fax: 0086-25-58731193; e-mail: dryanlinzhang@gmail.com.

Table S1. The location, the numbers of the monitoring sites in each city and the annual mean of $PM_{2.5}$ concentrations in 190 cities of China. The cities are ordered from the highest to the lowest $PM_{2.5}$ concentrations.

Order	City	Province	Numbers of sites	Latitude	Longitude	Population (Million)	$PM_{2.5}$ (μg/m3)
1	Baoding	Hebei	6	38.9	115.5	11.4	118.8
2	Xingtai	Hebei	4	37.1	114.5	7.2	109.6
3	Shijiazhuang	Hebei	8	38.0	114.5	10.5	101.4
4	Handan	Hebei	4	36.6	114.5	9.3	100.0
5	Dezhou	Shandong	3	37.5	116.3	5.6	97.6
6	Hengshui	Hebei	3	37.7	115.7	4.4	96.5
7	Liaocheng	Shandong	3	36.5	116.0	5.8	93.9
8	Zhengzhou	Henan	9	34.8	113.7	9.2	93.2
9	Heze	Shandong	3	35.2	115.5	8.3	93.0
10	Laiwu	Shandong	3	36.2	117.7	1.3	91.8
11	Tangshan	Hebei	6	39.6	118.0	7.7	91.5
12	Zibo	Shandong	6	36.8	118.1	4.6	89.7
13	Anyang	Henan	5	36.1	114.4	5.1	89.6
14	Langfang	Hebei	4	39.5	116.7	4.5	87.8
15	Linyi	Shandong	4	35.1	118.4	10.1	85.8
16	Jiaozuo	Henan	4	35.2	113.2	3.5	84.8
17	Zaozhuang	Shandong	5	34.9	117.6	3.8	84.6
18	Jinan	Shandong	8	36.7	117.0	6.9	84.6
19	Yichang	Hubei	5	30.7	111.3	4.1	84.4
20	Shouguang	Shandong	2	36.9	118.7	1.1	84.0
21	Binzhou	Shandong	3	37.4	118.0	3.8	80.4
22	Beijing	Beijing	12	39.9	116.5	21.1	79.8
23	Tianjin	Tianjin	15	39.1	117.2	14.7	79.7
24	Jining	Shandong	3	35.4	116.6	8.1	79.1
25	Pingdingshan	Henan	4	33.8	113.3	5.0	79.0
26	Cangzhou	Hebei	3	38.3	116.8	7.3	78.5
27	Jingzhou	Hubei	3	30.3	112.2	5.7	77.8
28	Kaifeng	Henan	4	34.8	114.4	4.6	74.8
29	Shenyang	Liaoning	11	41.8	123.4	7.3	74.1
30	Wuhan	Hubei	10	30.5	114.3	10.2	73.3
31	Hefei	Anhui	10	31.9	117.3	7.6	73.2
32	Dongying	Shandong	4	37.5	118.5	2.1	72.9
33	Zigong	Sichuan	4	29.4	104.8	2.7	72.6
34	Weifang	Shandong	5	36.6	119.1	9.2	72.6
35	Harbin	Heilongjiang	12	45.8	126.6	10.6	72.1

Order	City	Province	Numbers of sites	Latitude	Longitude	Population (Million)	$PM_{2.5}$ (μg/m3)
36	Sanmenxia	Henan	4	34.8	111.2	2.2	72.0
37	Jiangyin	Jiangsu	3	31.9	120.3	1.2	71.2
38	Korla	Xinjiang	3	41.7	86.1	0.5	70.6
39	Tai'an	Shandong	3	36.2	117.1	5.5	70.2
40	Luoyang	Henan	7	34.7	112.4	6.6	69.5
41	Anshan	Liaoning	7	41.1	122.9	3.5	69.2
42	Changsha	Hunan	10	28.2	113.0	7.1	68.3
43	Taizhou	Jiangsu	4	32.5	119.9	4.6	68.3
44	Nanjing	Jiangsu	9	32.0	118.8	8.1	67.8
45	Wuxi	Jiangsu	8	31.6	120.3	6.4	66.4
46	Changzhou	Jiangsu	6	31.8	120.0	4.7	66.4
47	Zhangjiagang	Jiangsu	2	31.9	120.6	0.9	66.0
48	Zhuji	Zhejiang	2	29.7	120.2	1.1	65.1
49	Xuzhou	Jiangsu	7	34.3	117.2	8.6	64.7
50	Suqian	Jiangsu	4	34.0	118.3	4.8	64.5
51	Changchun	Jilin	10	43.9	125.4	7.6	64.4
52	Suzhou	Jiangsu	8	31.3	120.6	10.5	64.4
53	Zhuzhou	Hunan	7	27.8	113.2	3.9	64.3
54	Jurong	Jiangsu	2	32.0	119.2	0.6	64.1
55	Urumchi	Xinjiang	7	43.8	87.7	3.3	64.1
56	Xiangtan	Hunan	7	27.9	112.9	2.8	63.6
57	Taiyuan	Shanxi	9	37.9	112.5	4.3	62.8
58	Maanshan	Anhui	5	31.6	118.5	2.2	62.6
59	Huai'an	Jiangsu	5	33.5	119.2	4.8	62.5
60	Chengdu	Sichuan	8	30.7	104.1	14.3	62.2
61	Zhenjiang	Jiangsu	4	32.2	119.4	3.1	61.9
62	Linfen	Shanxi	6	36.1	111.5	4.4	61.8
63	Changzhi	Shanxi	5	36.2	113.1	3.4	61.5
64	Wafangdian	Liaoning	2	39.6	122.0	1.0	61.2
65	Nanchong	Sichuan	6	0.0	106.1	6.3	61.1
66	Liuzhou	Guangxi	6	24.3	109.4	3.8	60.7
67	Tongchuan	Shaanxi	4	35.1	109.1	0.8	60.6
68	Yangquan	Shanxi	6	37.9	113.6	1.4	60.3
69	Yibin	Sichuan	6	29.8	104.6	4.5	60.2
70	Wuhu	Anhui	4	31.3	118.4	3.6	60.2
71	Yangzhou	Jiangsu	4	32.4	119.4	4.5	59.8
72	Zhangqiu	Shandong	2	36.7	117.5	1.0	59.8
73	Nantong	Jiangsu	5	32.0	120.9	7.3	59.8

Order	City	Province	Numbers of sites	Latitude	Longitude	Population (Million)	$PM_{2.5}$ (μg/m3)
74	Luzhou	Sichuan	4	28.9	105.4	4.2	59.7
75	Rizhao	Shandong	3	35.4	119.5	2.8	59.6
76	Huzhou	Zhejiang	3	30.9	120.1	2.9	59.5
77	Yiwu	Zhejiang	2	29.3	120.1	0.8	59.3
78	Xi'an	Shaanxi	13	34.3	109.0	8.6	59.2
79	Chongqing	Chongqing	17	29.6	106.5	29.7	59.1
80	Hangzhou	Zhejiang	11	30.3	120.2	8.8	59.0
81	Liyang	Jiangsu	2	31.4	119.5	0.8	59.0
82	Taicang	Jiangsu	2	31.5	121.1	0.5	58.9
83	Laixi	Shandong	2	36.9	120.5	0.7	58.9
84	Jinzhou	Liaoning	5	41.1	121.2	3.1	58.8
85	Linan	Zhejiang	2	30.2	119.7	0.5	58.7
86	Shaoxing	Zhejiang	3	30.0	120.6	4.9	58.5
87	Wujiang	Jiangsu	3	31.2	120.6	0.8	58.2
88	Fuyang	Zhejiang	2	30.1	120.0	0.7	58.1
89	Laizhou	Shandong	2	37.2	119.9	0.9	57.9
90	Pingdu	Shandong	2	36.8	120.0	1.4	57.9
91	Jinhua	Zhejiang	3	29.1	119.6	5.4	57.8
92	Weinan	Shaanxi	4	34.5	109.5	5.3	57.8
93	Jiaonan	Shandong	2	35.9	120.0	0.8	57.8
94	Fushun	Liaoning	6	42.0	124.0	2.2	57.6
95	Xining	Qinghai	4	36.6	101.7	2.2	57.1
96	Qinhuangdao	Hebei	5	40.0	119.6	3.0	57.0
97	Jilin	Jilin	7	43.9	126.6	4.3	56.8
98	Yixing	Jiangsu	2	31.4	119.8	1.1	56.6
99	Guilin	Guangxi	4	25.3	110.3	4.8	56.6
100	Xianyang	Shaanxi	4	34.4	108.7	4.9	56.0
101	Panjin	Liaoning	3	41.1	122.1	1.3	56.0
102	Lianyungang	Jiangsu	4	34.6	119.2	4.4	55.7
103	Changde	Hunan	5	29.1	111.7	5.7	55.7
104	Lanzhou	Gansu	5	36.0	103.7	3.6	55.6
105	Changshu	Jiangsu	3	31.6	120.7	1.1	55.5
106	Haimen	Jiangsu	2	31.9	121.2	1.0	55.3
107	Benxi	Liaoning	6	41.3	123.7	1.5	55.1
108	Jiaxing	Zhejiang	3	30.8	120.8	4.6	54.8
109	Zhaoyuan	Shandong	2	37.4	120.4	0.6	54.7
110	Jintan	Jiangsu	2	31.7	119.6	0.6	54.2
111	Huludao	Liaoning	4	40.7	120.9	2.8	53.7
112	Baoji	Shaanxi	8	34.4	107.2	3.7	53.6

Order	City	Province	Numbers of sites	Latitude	Longitude	Population (Million)	$PM_{2.5}$ (μg/m3)
113	Shanghai	Shanghai	10	31.2	121.5	24.2	53.0
114	Zhangjiajie	Hunan	4	29.1	110.5	1.5	52.7
115	Yancheng	Jiangsu	4	33.4	120.1	7.2	52.7
116	Jiaozhou	Shandong	2	36.3	120.0	0.8	52.4
117	Qingdao	Shandong	9	36.1	120.3	8.8	52.1
118	Mudanjiang	Heilongjiang	5	44.6	129.6	2.8	52.0
119	Yueyang	Hunan	6	29.4	113.1	5.5	51.8
120	Deyang	Sichuan	4	31.1	104.4	3.5	51.6
121	Zunyi	Guizhou	5	27.7	106.9	6.1	51.5
122	Kunshan	Jiangsu	2	31.4	121.0	0.7	50.7
123	Jimo	Shandong	2	36.4	120.5	1.1	50.5
124	Dalian	Liaoning	10	38.9	121.6	5.9	48.8
125	Baotou	Inner Mongolia	6	40.6	110.0	2.8	48.7
126	Dandong	Liaoning	4	40.1	124.4	2.4	48.7
127	Yantai	Shandong	6	37.5	121.4	7.0	47.8
128	Chengde	Hebei	5	41.0	117.9	3.5	47.6
129	Chaozhou	Guangdong	3	23.7	116.6	2.7	47.3
130	Quzhou	Zhejiang	3	29.0	118.9	2.1	47.1
131	Rushan	Shandong	2	36.9	121.5	0.6	46.9
132	Yinchuan	Ningxia	6	38.5	106.3	2.1	46.5
133	Nanchang	Jiangxi	9	28.7	115.9	5.1	46.5
134	Ningbo	Zhejiang	8	29.9	121.6	7.7	46.3
135	Jinyang	Sichuan	4	31.5	104.7	4.7	46.2
136	Yan'an	Shaanxi	4	36.6	109.5	2.2	45.7
137	Taizhou	Zhejiang	3	28.6	121.4	6.0	45.3
138	Wenzhou	Zhejiang	4	28.0	120.7	9.2	45.3
139	Zhaoqing	Guangdong	4	23.1	112.4	4.0	45.1
140	Penglai	Shandong	2	37.8	120.8	0.5	45.0
141	Nanning	Guangxi	8	22.8	108.3	6.9	45.0
142	Shizuishan	Ningxia	4	39.0	106.4	0.8	44.9
143	Wendeng	Shandong	2	37.2	122.1	0.6	44.1
144	Guiyang	Guizhou	11	26.6	106.7	4.4	42.9
145	Jieyang	Guangdong	4	23.6	116.4	6.0	42.9
146	Jinchang	Gansu	3	38.5	102.2	0.5	42.8
147	Lishui	Zhejiang	3	28.5	119.9	2.1	42.6
148	Yingkou	Liaoning	4	40.7	122.2	2.3	42.4
149	Chifeng	Inner Mongolia	4	42.3	119.0	4.3	42.4

Order	City	Province	Numbers of sites	Latitude	Longitude	Population (Million)	$PM_{2.5}$ (μg/m3)
150	Guangzhou	Guangdong	11	23.2	113.2	12.9	42.4
151	Jiangmen	Guangdong	4	22.6	113.1	4.5	41.5
152	Jiujiang	Jiangxi	8	29.7	116.0	5.0	41.4
153	Hohhot	Inner Mongolia	8	40.8	111.7	3.0	41.2
154	Daqing	Heilongjiang	5	46.6	125.1	2.9	41.0
155	Foshan	Guangdong	8	23.1	113.1	7.3	40.8
156	Shaoguan	Guangdong	5	24.8	113.6	2.9	40.1
157	Datong	Shanxi	6	40.1	113.3	3.4	39.8
158	Dongguan	Guangdong	5	23.0	113.8	8.3	39.2
159	Jiayuguan	Gansu	2	39.8	98.2	0.2	38.7
160	Rongcheng	Shandong	2	37.2	122.4	0.7	38.6
161	Weihai	Shandong	3	37.5	122.1	2.8	38.4
162	Meizhou	Guangdong	3	24.6	116.1	4.3	38.0
163	Heyuan	Guangdong	3	23.7	114.7	3.0	37.9
164	Qingyuan	Guangdong	3	23.7	113.0	3.8	36.7
165	Shantou	Guangdong	6	23.4	116.7	5.5	36.0
166	Qiqihar	Heilongjiang	5	47.3	123.9	5.4	35.8
167	Zhongshan	Guangdong	4	22.5	113.4	3.2	35.6
168	Maoming	Guangdong	4	21.7	110.9	6.0	35.2
169	Panzhihua	Sichuan	5	26.6	101.7	1.2	35.1
170	Karamay	Xinjiang	5	45.6	84.8	0.7	34.8
171	Yangjiang	Guangdong	3	21.9	112.0	2.5	34.4
172	Xiamen	Fujian	4	24.5	118.1	3.6	34.1
173	Zhuhai	Guangdong	4	22.3	113.5	1.6	33.8
174	Qujing	Yunnan	2	25.5	103.8	6.3	32.9
175	Yunfu	Guangdong	3	22.9	112.0	2.4	32.4
176	Shenzhen	Guangdong	11	22.6	114.1	10.6	32.0
177	Kunming	Yunnan	7	25.0	102.7	5.4	31.9
178	Fuzhou	Fujian	6	26.1	119.3	7.2	31.8
179	Shanwei	Guangdong	3	22.8	115.4	3.0	30.9
180	Huizhou	Guangdong	5	23.1	114.4	4.7	30.4
181	Beihai	Guangxi	4	21.5	109.1	1.6	30.2
182	Quanzhou	Fujian	4	24.9	118.6	8.2	30.1
183	Zhoushan	Zhejiang	3	30.0	122.1	1.1	30.0
184	Yuxi	Yunnan	3	24.4	102.5	2.3	28.8
185	Zhanjiang	Guangdong	6	21.2	110.4	7.2	27.9
186	Zhangjiakou	Hebei	5	40.8	114.9	4.4	27.1

Order	City	Province	Numbers of sites	Latitude	Longitude	Population (Million)	$PM_{2.5}$ (μg/m3)
187	Ordos	Inner Mongolia	5	39.8	110.0	2.0	26.5
188	Lhasa	Tibet	6	30.0	91.1	0.5	22.8
189	Haikou	Hainan	5	20.0	110.4	2.2	21.7
190	Sanya	Hainan	2	18.3	109.5	0.7	17.7

Table S2. $PM_{2.5}$ and PM_{10} concentrations (μg/m^3) in each season in 2014/2015 in 190 cities of China.

City	$PM_{2.5}$					PM_{10}				
	Yearly mean	Spring	Summer	Fall	Winter	Yearly mean	Spring	Summer	Fall	Winter
Baoding	118.8	89.0	78.3	136.1	185.4	203.8	183.8	136.5	227.2	278.0
Xingtai	109.6	94.2	77.9	127.3	141.4	195.9	198.6	140.0	215.1	230.0
Shijiazhuang	101.4	90.8	83.4	109.8	126.6	173.2	171.5	141.4	179.3	206.2
Handan	100.0	82.8	80.5	113.2	133.5	174.8	163.4	135.2	188.8	221.0
Dezhou	97.6	85.4	77.5	103.8	127.7	150.5	150.5	113.6	149.5	188.2
Hengshui	96.5	79.7	75.8	109.9	130.4	181.0	176.6	131.0	185.7	236.6
Liaocheng	93.9	80.1	75.3	102.9	121.6	161.1	150.9	138.2	174.0	184.5
Zhengzhou	93.2	87.2	61.0	97.7	129.1	158.2	155.2	118.1	153.1	207.4
Heze	93.0	81.1	66.0	103.5	125.4	152.9	146.0	103.9	158.6	205.2
Laiwu	91.8	84.3	75.9	92.9	116.9	136.0	136.4	114.8	133.6	159.1
Tangshan	91.5	88.7	77.0	95.8	112.7	155.7	163.0	131.0	156.8	178.2
Zibo	89.7	86.1	74.1	92.0	107.6	163.4	165.3	135.5	162.2	189.9
Anyang	89.6	74.9	67.1	88.0	133.3	140.1	129.9	113.9	130.3	189.6
Langfang	87.8	77.3	69.2	104.8	110.1	142.2	142.1	112.9	161.6	163.5
Linyi	85.8	82.1	61.5	81.5	119.4	162.0	161.4	137.5	155.3	194.2
Jiaozuo	84.8	89.9	67.4	72.3	107.7	139.9	154.6	107.2	114.3	178.8
Zaozhuang	84.6	82.6	66.2	72.3	118.1	158.9	170.3	122.3	138.9	200.2
Jinan	84.6	81.3	73.2	86.7	98.1	164.4	170.8	131.5	165.9	187.3
Yichang	84.4	72.6	57.5	72.7	138.8	126.3	114.4	96.0	112.8	186.1
Shouguang	84.0	81.0	70.8	79.7	104.9	143.6	161.4	114.3	130.4	168.6
Binzhou	80.4	79.4	66.0	80.3	96.3	129.3	129.8	107.4	133.9	145.9
Beijing	79.8	78.2	68.0	90.4	81.4	110.2	119.7	87.2	120.8	114.4
Tianjin	79.7	72.7	63.4	88.4	106.8	129.1	130.0	102.4	129.4	157.3

Jining	79.1	70.7	73.5	79.7	95.2	141.8	143.3	106.6	139.0	177.8
Pingdingshan	79.0	72.3	58.2	79.0	108.5	131.9	125.8	99.1	125.6	179.1
Cangzhou	78.5	72.5	66.2	85.8	101.5	137.2	131.4	107.1	150.8	172.8
Jingzhou	77.8	67.8	60.7	76.4	109.6	125.9	115.4	102.4	125.1	164.2
Kaifeng	74.8	66.3	59.5	81.4	95.0	124.1	127.7	95.7	125.9	145.8
Shenyang	74.1	63.5	49.7	86.7	100.1	123.3	113.1	87.0	142.5	154.2
Wuhan	73.3	61.2	56.8	71.2	107.9	104.9	98.4	93.8	103.5	126.0
Hefei	73.2	66.3	68.4	74.6	85.7	104.0	95.8	101.3	111.5	110.1
Dongying	72.9	71.1	60.0	73.0	88.0	137.2	154.4	118.9	130.2	139.4
Zigong	72.6	62.5	45.2	64.1	121.9	109.8	102.7	80.6	95.0	163.4
Weifang	72.6	65.5	57.5	80.2	89.4	139.8	143.8	111.7	142.1	160.2
Harbin	72.1	47.7	35.9	100.0	112.9	108.1	87.2	62.6	137.7	152.0
Sanmenxia	72.0	71.4	57.9	69.9	88.8	125.7	134.0	100.4	116.9	148.9
Jiangyin	71.2	72.5	61.2	59.5	91.6	106.8	108.8	90.5	91.9	136.0
Korla	70.6	108.4	38.1	58.4	64.9	252.8	509.4	112.1	176.8	127.6
Tai'an	70.2	59.8	60.0	71.3	93.1	126.9	117.9	101.0	124.5	167.2
Luoyang	69.5	73.5	46.3	68.3	88.7	120.7	132.9	91.5	108.2	145.9
Anshan	69.2	60.7	56.4	79.9	82.5	117.6	112.2	93.2	133.8	133.1
Changsha	68.3	51.6	55.7	74.4	97.2	84.6	74.3	80.8	92.5	94.4
Taizhou	68.3	62.5	65.4	61.2	86.1	105.9	105.3	90.0	93.2	135.2
Nanjing	67.8	62.6	65.1	66.5	78.8	112.7	110.7	92.9	111.5	136.2
Wuxi	66.4	63.9	55.5	61.1	86.2	103.1	101.0	83.5	98.5	130.0
Changzhou	66.4	64.8	55.5	57.5	88.3	103.0	104.5	83.7	93.9	129.6
Zhangjiagang	66.0	61.1	52.7	58.4	91.9	118.4	105.7	98.9	119.0	150.0
Zhuji	65.1	79.4	88.7	56.9	40.1	98.6	136.0	106.4	91.8	72.8
Xuzhou	64.7	65.3	51.8	48.8	92.8	120.3	126.4	86.6	102.8	163.1
Suqian	64.5	64.3	61.7	51.2	81.1	111.0	115.1	99.8	90.8	137.0

Changchun	64.4	46.9	42.6	83.0	91.1	110.1	96.3	86.7	137.3	124.6
Suzhou	64.4	62.7	56.2	55.7	83.7	87.8	84.7	78.3	80.4	108.8
Zhuzhou	64.3	50.5	50.9	66.2	94.2	96.7	82.4	74.7	99.5	135.0
Jurong	64.1	65.0	58.9	51.8	80.8	70.4	78.2	67.6	62.7	73.2
Urumchi	64.1	48.9	33.3	51.2	128.1	147.1	134.3	125.2	136.9	196.1
Xiangtan	63.6	49.1	53.8	68.6	87.7	101.3	87.5	81.2	108.2	132.9
Taiyuan	62.8	52.3	45.6	77.7	79.2	117.3	110.9	92.9	138.8	128.9
Maanshan	62.6	59.8	58.9	55.2	77.6	94.5	96.2	91.7	85.1	104.6
Huai'an	62.5	54.4	55.5	52.1	90.5	98.6	98.9	78.3	79.7	137.4
Chengdu	62.2	57.4	40.6	52.5	99.9	104.7	100.1	69.1	89.8	161.2
Zhenjiang	61.9	59.2	63.0	51.1	75.4	104.4	104.7	99.6	96.6	116.7
Linfen	61.8	56.6	55.2	59.2	77.8	95.4	99.8	80.4	84.6	115.4
Changzhi	61.5	48.2	47.5	78.1	76.7	106.9	105.3	87.5	115.0	120.3
Wafangdian	61.2	56.8	49.6	68.2	70.9	74.9	86.9	64.3	80.9	75.8
Nanchong	61.1	51.8	47.3	50.9	97.7	93.1	86.6	72.5	77.0	138.7
Liuzhou	60.7	48.9	42.7	69.2	86.2	86.7	69.1	66.6	103.7	113.2
Tongchuan	60.6	59.8	41.2	61.6	80.0	113.2	120.6	80.3	107.0	142.5
Yangquan	60.3	55.2	52.5	72.7	62.4	136.3	133.2	115.5	161.3	136.2
Yibin	60.2	57.3	36.1	48.2	100.3	89.4	91.0	57.6	71.3	137.4
Wuhu	60.2	57.4	63.7	48.3	72.5	89.7	94.3	96.0	64.6	102.6
Yangzhou	59.8	62.0	64.1	41.4	71.1	108.9	110.7	107.5	82.3	134.6
Zhangqiu	59.8	64.2	51.1	49.2	75.0	106.7	126.3	72.0	84.8	143.7
Nantong	59.8	56.5	51.1	51.6	81.2	91.3	88.4	71.8	81.8	124.0
Luzhou	59.7	55.2	39.2	45.3	100.5	88.4	84.8	57.8	66.6	145.8
Rizhao	59.6	56.7	43.9	56.2	82.3	107.2	113.8	76.6	100.2	136.2
Huzhou	59.5	55.5	46.0	54.8	83.3	79.2	75.2	66.9	77.7	98.4
Yiwu	59.3	61.1	44.4	49.2	83.3	93.8	91.5	72.1	79.1	133.1

Xi'an	59.2	54.1	41.8	66.6	76.2	128.5	130.4	92.3	127.0	163.8
Chongqing	59.1	42.2	33.2	65.7	100.8	90.5	73.3	59.6	98.8	136.3
Hangzhou	59.0	54.4	47.5	59.9	75.8	91.0	88.5	76.7	91.9	107.8
Liyang	59.0	64.3	53.5	46.1	72.0	97.2	106.7	79.9	81.0	121.0
Taicang	58.9	59.3	55.7	42.2	79.4	88.3	96.1	79.9	65.6	115.4
Laixi	58.9	56.8	42.6	54.8	81.8	87.8	92.9	59.2	84.3	114.8
Jinzhou	58.8	61.3	48.1	63.1	62.1	96.1	105.0	72.6	103.9	99.8
Linan	58.7	65.7	53.1	46.6	72.2	101.9	114.1	99.8	77.6	122.1
Shaoxing	58.5	52.4	47.3	61.6	74.8	86.5	81.1	59.1	90.8	117.0
Wujiang	58.2	56.9	55.5	45.8	76.4	95.8	93.7	94.5	80.0	118.9
Fuyang	58.1	53.2	53.1	52.2	71.9	101.8	97.0	81.6	85.8	140.5
Laizhou	57.9	58.2	46.8	55.0	71.9	108.6	126.5	90.6	96.1	121.2
Pingdu	57.9	58.2	48.8	52.5	72.2	89.7	108.0	67.3	80.0	103.8
Jinhua	57.8	52.7	46.5	61.8	71.9	81.1	70.7	60.7	88.6	107.8
Weinan	57.8	53.2	40.7	67.0	71.7	110.0	119.8	83.3	113.7	120.1
Jiaonan	57.8	54.1	43.0	54.3	79.9	89.7	80.3	56.4	88.7	122.2
Fushun	57.6	46.3	44.2	63.2	80.6	104.6	92.6	75.8	119.8	134.1
Xining	57.1	58.4	47.7	57.6	64.1	114.3	152.4	81.2	94.8	116.1
Qinhuangdao	57.0	57.6	38.4	61.0	73.7	113.0	125.9	72.1	117.8	140.3
Jilin	56.8	39.4	34.0	75.5	84.1	95.9	79.0	63.0	120.4	126.7
Yixing	56.6	58.3	55.0	45.1	70.3	80.6	79.9	80.0	63.7	100.9
Guilin	56.6	46.9	41.2	64.9	76.6	77.2	66.5	59.4	85.9	100.7
Xianyang	56.0	52.0	36.9	64.7	72.0	111.3	111.9	83.5	114.6	135.2
Panjin	56.0	53.9	47.2	56.9	66.6	85.9	88.7	65.1	87.5	101.4
Lianyungang	55.7	49.4	47.6	41.7	86.3	103.9	105.5	86.9	87.3	135.3
Changde	55.7	46.5	51.9	56.8	70.8	85.2	78.3	73.7	87.1	103.9
Lanzhou	55.6	55.0	46.9	54.2	66.4	123.7	161.5	93.0	101.3	126.2

Changshu	55.5	59.6	48.9	41.0	74.6	82.2	83.8	70.2	62.5	111.9
Haimen	55.3	52.2	42.6	45.1	77.6	84.6	87.7	55.6	70.4	119.6
Benxi	55.1	43.8	42.6	64.8	72.9	96.2	80.8	74.6	116.9	117.7
Jiaxing	54.8	51.5	44.2	49.9	74.8	80.6	78.4	63.0	72.6	109.1
Zhaoyuan	54.7	58.7	46.4	51.4	62.4	93.7	110.7	71.7	87.0	105.5
Jintan	54.2	63.8	50.8	37.3	70.9	83.3	97.9	83.7	62.4	100.3
Huludao	53.7	56.2	47.5	45.4	64.9	107.8	112.9	84.9	105.9	125.7
Baoji	53.6	54.4	36.8	55.9	67.1	102.1	115.4	70.7	101.6	116.1
Shanghai	53.0	53.7	42.1	42.5	73.6	73.3	70.8	57.5	63.5	102.1
Zhangjiajie	52.7	46.3	41.7	47.8	77.2	80.3	72.1	61.6	75.7	114.7
Yancheng	52.7	47.4	44.6	43.9	76.7	88.2	86.3	73.1	72.8	121.2
Jiaozhou	52.4	50.2	48.4	48.8	62.4	84.7	91.9	70.5	84.0	92.3
Qingdao	52.1	51.1	40.2	48.0	69.6	102.7	107.2	82.9	96.8	122.4
Mudanjiang	52.0	41.1	37.7	63.6	69.3	84.7	73.0	68.0	102.1	99.7
Yueyang	51.8	44.5	45.7	51.9	67.5	111.3	93.7	102.7	114.8	140.0
Deyang	51.6	51.2	40.3	38.7	76.5	84.3	91.2	69.3	60.6	113.7
Zunyi	51.5	48.5	41.8	48.6	68.1	83.5	81.8	70.9	79.0	103.1
Kunshan	50.7	57.7	46.3	34.6	67.4	86.2	95.3	83.5	63.2	106.6
Jimo	50.5	40.4	38.5	53.1	70.4	89.7	85.3	61.1	96.8	116.0
Dalian	48.8	48.4	42.9	49.0	54.9	82.6	90.0	66.2	80.5	91.2
Baotou	48.7	41.1	35.4	54.3	66.6	131.5	141.2	97.1	141.0	143.6
Dandong	48.7	50.7	35.7	44.0	63.7	84.6	88.2	58.5	78.5	112.0
Yantai	47.8	50.3	43.8	41.7	54.7	78.1	89.1	70.3	67.2	81.9
Chengde	47.6	43.4	44.0	55.9	50.2	100.0	102.8	79.9	122.0	106.0
Chaozhou	47.3	45.6	35.4	51.8	57.1	58.9	58.2	41.2	58.5	77.8
Quzhou	47.1	44.6	39.1	47.2	58.2	76.5	72.0	59.0	76.9	99.6
Rushan	46.9	42.7	34.3	47.9	63.1	63.6	66.5	45.7	58.9	83.7

Yinchuan	46.5	40.3	33.8	50.3	63.9	109.5	124.2	75.7	100.3	132.6
Nanchang	46.5	37.6	36.9	58.5	56.0	74.5	65.7	66.6	87.5	81.1
Ningbo	46.3	42.0	29.9	42.2	72.3	73.2	67.2	47.7	68.4	111.3
Jinyang	46.2	44.4	39.5	34.9	66.6	71.6	75.0	58.3	54.7	97.1
Yan'an	45.7	43.6	34.7	45.6	59.8	112.5	121.2	90.9	106.3	128.7
Taizhou	45.3	43.0	31.5	42.1	65.5	68.6	63.5	46.9	65.3	100.3
Wenzhou	45.3	45.9	32.1	41.8	61.3	73.8	76.0	53.0	68.0	97.5
Zhaoqing	45.1	39.2	32.0	54.8	56.5	69.1	59.6	56.9	80.3	82.9
Penglai	45.0	46.9	35.5	46.1	51.7	73.4	95.8	59.5	63.2	74.6
Nanning	45.0	35.2	24.1	50.9	73.0	76.8	61.9	52.3	86.7	111.4
Shizuishan	44.9	46.3	28.8	46.6	58.0	124.0	151.6	73.5	119.4	151.4
Wendeng	44.1	39.3	39.7	44.1	53.7	65.5	74.8	51.2	55.4	80.7
Guiyang	42.9	40.7	29.3	40.4	61.8	67.7	70.3	51.2	62.9	85.4
Jieyang	42.9	46.5	34.8	40.3	48.6	61.8	68.5	52.1	60.9	63.4
Jinchang	42.8	60.4	21.9	35.0	48.0	124.3	200.3	63.5	91.0	117.2
Lishui	42.6	37.9	28.0	45.5	60.4	63.8	54.5	44.9	70.9	88.0
Yingkou	42.4	46.4	30.3	37.1	54.5	71.8	83.7	50.2	64.5	85.1
Chifeng	42.4	35.1	32.7	47.6	56.6	97.9	98.0	75.8	116.5	101.4
Guangzhou	42.4	33.9	33.3	47.8	57.3	62.6	51.3	52.4	69.3	81.3
Jiangmen	41.5	30.8	23.1	49.5	65.9	61.2	50.5	40.4	66.6	90.9
Jiujiang	41.4	33.2	37.7	45.6	52.0	77.2	65.4	69.1	91.7	86.5
Hohhot	41.2	34.6	27.6	43.5	61.2	112.3	115.0	91.0	117.0	125.6
Daqing	41.0	35.2	32.5	39.1	59.0	62.1	62.0	50.3	58.9	77.3
Foshan	40.8	32.0	28.4	49.4	56.3	60.3	48.8	45.5	70.1	80.8
Shaoguan	40.1	29.9	32.8	52.6	48.3	59.6	44.8	52.6	76.1	70.1
Datong	39.8	37.0	34.1	33.2	55.6	94.2	110.7	66.7	72.9	121.1
Dongguan	39.2	29.5	28.0	45.8	56.9	55.8	42.7	42.4	65.7	76.9

Jiayuguan	38.7	55.5	27.0	33.0	33.6	133.4	196.3	87.0	118.0	111.2
Rongcheng	38.6	42.3	36.2	32.7	43.5	66.6	81.0	53.4	58.5	73.5
Weihai	38.4	41.5	36.1	33.3	41.5	71.7	81.4	60.6	63.5	78.1
Meizhou	38.0	33.3	24.7	45.9	49.5	58.5	49.8	45.9	72.6	68.6
Heyuan	37.9	29.2	31.3	43.7	50.5	56.1	42.5	46.1	66.5	73.9
Qingyuan	36.7	33.6	22.0	40.0	52.1	58.5	55.1	41.4	63.1	75.3
Shantou	36.0	33.2	21.6	39.3	50.8	59.2	52.6	41.1	66.6	78.6
Qiqihar	35.8	29.5	25.0	42.5	48.1	62.1	62.9	50.3	65.1	69.8
Zhongshan	35.6	26.8	19.6	42.7	56.3	52.8	39.7	30.8	64.1	81.2
Maoming	35.2	22.8	18.8	42.5	60.8	53.1	38.6	34.0	63.4	81.3
Panzhihua	35.1	38.6	31.5	25.2	44.0	72.8	76.4	70.3	53.8	89.5
Karamay	34.8	26.7	27.5	30.4	57.4	71.9	63.2	60.7	66.8	99.8
Yangjiang	34.4	25.8	16.4	45.3	52.9	52.2	38.7	29.9	66.5	78.0
Xiamen	34.1	32.8	26.9	33.8	43.2	54.3	52.1	46.0	55.6	64.2
Zhuhai	33.8	23.5	14.5	40.2	60.6	54.6	38.7	34.1	67.8	83.2
Qujing	32.9	36.8	26.0	32.3	35.0	54.1	63.2	39.4	54.1	56.8
Yunfu	32.4	23.6	21.0	44.0	43.8	52.8	39.8	36.7	66.8	72.4
Shenzhen	32.0	24.8	18.2	38.1	49.5	55.3	43.7	40.2	64.5	76.6
Kunming	31.9	39.7	21.7	28.4	35.1	61.2	74.2	44.0	60.1	62.0
Fuzhou	31.8	32.7	23.6	28.9	41.6	61.5	63.2	54.5	60.1	67.9
Shanwei	30.9	26.5	15.8	36.5	46.4	44.1	38.0	27.2	49.3	63.9
Huizhou	30.4	23.0	22.0	36.9	42.0	54.2	43.1	41.6	62.1	73.8
Beihai	30.2	21.4	9.0	35.1	58.2	54.1	45.5	33.7	60.1	80.0
Quanzhou	30.1	29.1	26.7	27.8	37.0	58.8	60.6	62.9	51.9	59.2
Zhoushan	30.0	29.4	21.8	23.8	45.1	49.5	46.6	31.2	43.7	77.4
Yuxi	28.8	38.5	18.3	23.7	31.3	49.9	66.5	34.2	41.8	51.5
Zhanjiang	27.9	20.4	14.6	34.3	44.8	47.6	36.4	30.4	57.8	69.7

Zhangjiakou	27.1	31.3	20.6	25.0	31.3	62.4	85.0	39.4	49.1	66.4
Ordos	26.5	26.5	21.9	27.0	30.6	71.8	89.0	48.6	63.1	80.8
Lhasa	22.8	23.0	16.5	23.9	27.9	53.4	59.1	39.4	55.8	57.2
Haikou	21.7	15.4	11.7	24.3	37.6	39.1	31.1	26.8	43.3	57.9
Sanya	17.7	15.2	9.6	19.4	27.6	33.5	28.0	24.4	38.5	45.0

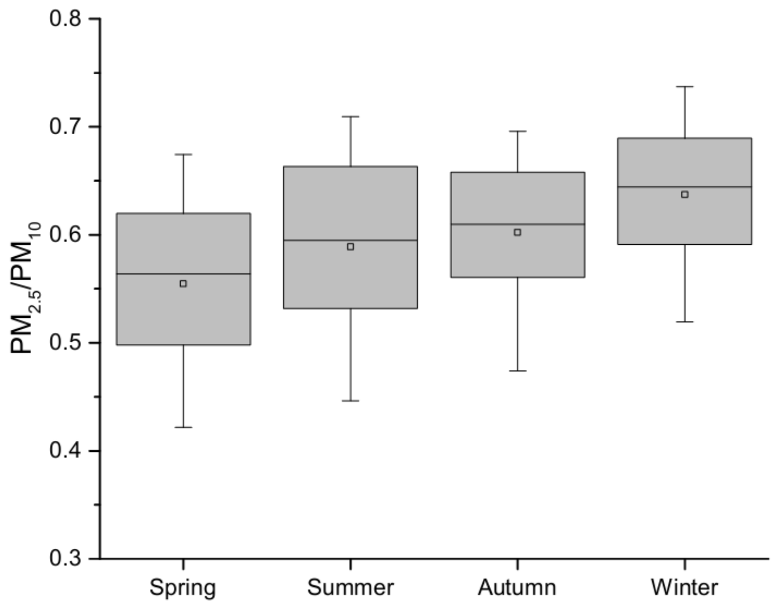

Figure S1. Box plot of $PM_{2.5}/PM_{10}$ ratio in different seasons. Each box shows the median (black line), mean (square), the interquartile range (box) and the 10th percentile and 90th percentile (the whiskers).

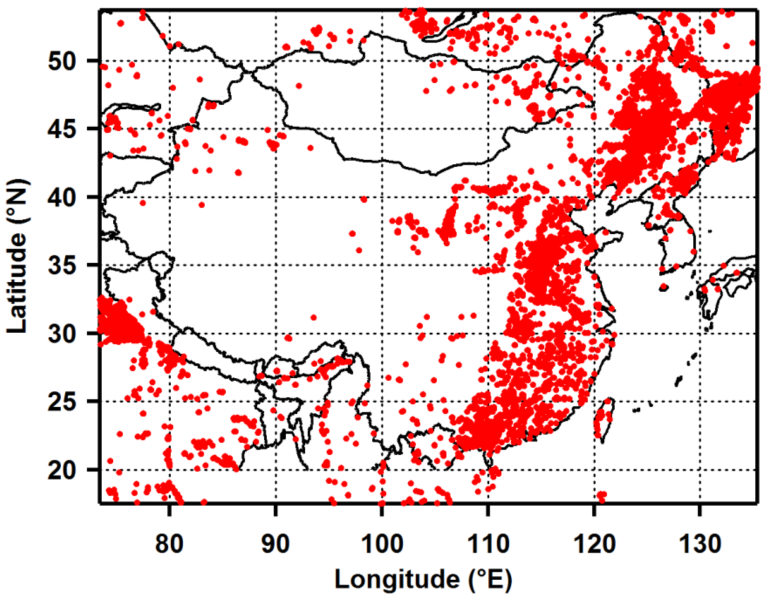

Figure S2. MODIS fire counts during the period from the October of 2014. Fire locations were obtained from produced by the University of Maryland and provided by NASA FIRMS operated by NASA/GSFC/ESDIS with funding provided by NASA/HQ (Available on-line https://earthdata.nasa.gov/active-fire-data#tab-content-6). The map was drawn by the software of Igor Pro, http://www.wavemetrics.com/.

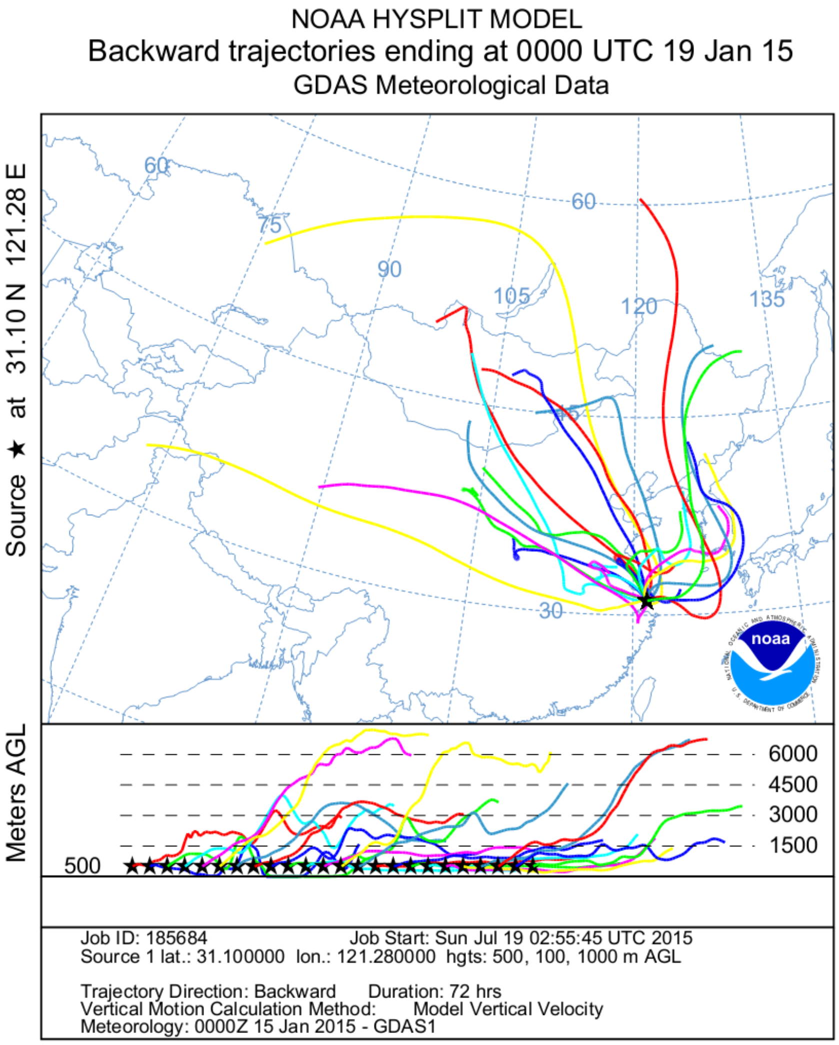

Figure S3. A typical 72-hour backward trajectory of air mass arriving Shanghai during winter. The trajectories were drawn using Hybrid Single Particle Lagrangian Integrated Trajectory (HYSPLIT) Model with the software HYSPLIT-WEB (http://ready.arl.noaa.gov/HYSPLIT.php) [1, 2].

References

1. Draxler, R.R. and Rolph, G.D., 2015. HYSPLIT (HYbrid Single-Particle Lagrangian Integrated Trajectory) Model access via NOAA ARL READY Website

(http://ready.arl.noaa.gov/HYSPLIT.php). NOAA Air Resources Laboratory, Silver Spring, MD.

2. Rolph, G.D., 2015. Real-time Environmental Applications and Display sYstem (READY) Website (http://ready.arl.noaa.gov). NOAA Air Resources Laboratory, Silver Spring, MD.
